# Editorial: Behavioral Addictions, Risk-Taking, and Impulsive Choice

**DOI:** 10.3389/fnbeh.2022.924030

**Published:** 2022-05-09

**Authors:** Marco Bortolato, Gregory J. Madden

**Affiliations:** ^1^Department of Pharmacology and Toxicology, University of Utah, Salt Lake City, UT, United States; ^2^Department of Psychology, Utah State University, Logan, UT, United States

**Keywords:** impulsivity, risk taking, animal models, delay discounting, probability discounting, decision making, behavioral addiction

This special topic presents theoretical and experimental work on the biopsychological mechanisms of impulsivity. While impulsivity is regarded as a core symptom in various psychiatric disorders, ranging from ADHD to disruptive disorders and behavioral addiction, current frameworks indicate that impulsivity is a multidimensional construct, which is currently interpreted as a cluster of different behavioral domains that likely reflect separate neurobiological mechanisms (Strickland and Johnson, 2020).

One of the main facets of impulsivity is maladaptive decision making, whereby immediate benefits (such as the rewarding effect of a drug or alcohol use or the escape/avoidance of physical/emotional pain) are preferred over more consequential, but delayed negative outcomes (e.g., health deficits, relationship loss). This devaluation of untoward consequences (i.e., steep delay-discounting) is borne out in the literature by meta-analyses showing robust (replicable, medium-to-large effect size) correlations between a variety of substance-use disorders and delay discounting (MacKillop et al., 2011; Amlung et al., 2017). In addition, several studies indicate that decreased delay discounting is associated with maladaptive health decision-making (e.g., Stein et al., 2016; Athamneh et al., 2021). Therefore, interventions that aim to decrease delay discounting are of some importance. The special topic paper by Stein et al. finds, for the first time, that a choice-bundling intervention reduces the extent to which cigarette smokers discount delayed gains *and* losses, the latter being analogous to loss of health, relationships, etc. Bundling interventions allow the decision-maker to make one choice and then experience a series of either smaller-sooner or larger-later rewards (depending on the initial choice). These interventions have proven effective in reducing delay discounting in human and non-human subjects (Rung and Madden, 2018; Smith et al., 2019), with the Stein et al. paper being the first to show the bundling strategy works to decrease the devaluation of delayed negative outcomes. The authors discuss bundling-based therapies that could help those at risk of substance use disorders to give greater consideration to the future outcomes of decisions made today.

Beyond interventions, there are several state-factors known to influence the rate of delay discounting (Odum et al., 2020). The special topic paper by Downey et al. reviews the human and non-human literature to evaluate if deprivation (e.g., hunger, thirst, acute drug withdrawal) is one such state variable that, when increased, increases impulsive choice. They find little uniformity in the literature, either in how deprivation is imposed (e.g., hypothetical vs. real deprivations of varying durations) or in the effect sizes these manipulations induce. They discuss the importance of better understanding deprivation effects, and how greater uniformity might be brought to the literature.

The special topic paper by Gilroy et al. examines the practice of excluding data because the shape of the discounting function is irregular, potentially reflecting inattention, or careless survey responding. To avoid the inadvertent exclusion of valid data, the authors explore a Latent Class Mixed Modeling approach, which classifies groups of obtained uncharacteristic patterns of choice. Their application of that approach to a publicly available dataset suggests it may prove a useful supplement to existing methods for screening out unsystematic discounting data. The paper by Grunevski et al. reveals that an independent measure of ambivalence systematically increases as participants complete survey questions that approach the point of subjective equivalence (i.e., when the smaller-sooner and larger-later outcomes are equally valued). Such measures of ambivalence are potentially useful in detecting (and excluding) data produced by careless participants, or in detecting shifting indifference points in interventions designed to reduce delay discounting.

Less is known about the correlation between delay discounting and maladaptive decision-making that does not involve substance use. The special topic paper by Weinsztok et al. provides a pre-registered systematic review and meta-analysis of 78 published studies evaluating delay discounting rates among those with a behavioral (non-substance) addiction. The clearest relation was observed among those with a gambling disorder, whereas other “addictions” (e.g., internet/smartphone, compulsive buying) have either not been adequately studied or are not consistently correlated with delay discounting. Concerns are raised about the potential for publication bias.

Gambling disorders are, unsurprisingly, also correlated with putting greater subjective value on probabilistic outcomes. The special topic paper by Schneider et al. replicates this finding in an American Indian sample of gamblers and non-gamblers. They also explore neural responses correlating with choices made in the probability discounting task. In a rat model of gambling, Vonder Haar et al. explore the effects of traumatic brain injury (TBI) on risky, suboptimal choices. They report that, despite considerable individual differences within groups, TBI rats were less sensitive to contingencies, less sensitive to recent outcomes, and demonstrated a general bias toward the riskier alternatives. Clustering the patterns of choice revealed distinct behavioral phenotypes, with TBI rats rarely demonstrating the optimal of these choice phenotypes.

Another critical dimension that can influence impulsivity is the sensitivity to environmental stress. In fact, ample evidence indicates that exposure to acute stress can modify decision making and promote the choice of rewarding options. Building on this idea, the article by Dong et al.  shows that tail-clip stress increases self-administration of propofol in rats through corticotropin-releasing factor (CRF) receptor 1, a key orchestrator of the stress response. These mechanisms, which are likely supported by dopamine 1 receptors in the nucleus accumbens, point to the crosstalk between CRF and mesolimbic dopamine neurotransmission as a key process shaping the negative influence of stress on drug seeking.

In addition to neuroeconomic alterations (such as those observed in delay and probability discounting), impulsivity is likely to encompass other constructs related to sensation-seeking, boredom susceptibility, and venturesomeness (Depue and Collins, 1999). However, operationalizing these dispositions, and identifying valid animal models that may appropriately capture their neurobiological foundations, has proven complex. In their article, Festucci et al. present a novel paradigm based on an adapted version of the suspended wire bridge protocol originally developed for mice (Bortolato et al., 2009). Using this behavioral task—which measures the propensity to engage in risky actions irrespective of rewards—the authors document that early-life exposure to adults with impaired dopamine reuptake reduces venturesome-like behavior.

Overall, we believe that the contributions to this Special Topic highlight the multifaceted nature of impulsivity and open to new empirical and theoretical perspectives in the definition of this complex behavioral construct. In closing, we would like to dedicate this Special Topic to the memory of Stephen C. Fowler, who passed away far too young in June 2020, and had dedicated his entire scientific life to behavioral pharmacology. As part of his extensive scientific legacy (attested in over 160 publications, many of which in high-impact, peer-reviewed journals, including *Science, Cell*, and *PNAS*), Steve developed novel quantitative methods for the detection and analysis of motor and cognitive responses. He provided major contributions to the research field of impulsivity and addiction by studying the impact of dopaminergic agonists in several animal models of risky choice and attention deficits. He was a brilliant scientist and innovator, and a staunch advocate of the essential value of animal models in neuropsychopharmacological and behavioral research. We both had the good luck to collaborate with him, and we will always remember him as a kind, open, and generous friend. We miss him deeply.

## Author Contributions

All authors listed have made a substantial, direct, and intellectual contribution to the work and approved it for publication.

## Funding

This work was funded by NIH (R21DA049530 to MB) and (R03DA052467 to GM).

## Conflict of Interest

The authors declare that the research was conducted in the absence of any commercial or financial relationships that could be construed as a potential conflict of interest.

## Publisher's Note

All claims expressed in this article are solely those of the authors and do not necessarily represent those of their affiliated organizations, or those of the publisher, the editors and the reviewers. Any product that may be evaluated in this article, or claim that may be made by its manufacturer, is not guaranteed or endorsed by the publisher.
